# Personalized Medicine in Coronary Artery Disease: Where Are We in 2022?

**DOI:** 10.3390/jpm12091446

**Published:** 2022-09-01

**Authors:** Dmitry Shchekochikhin, Philipp Kopylov

**Affiliations:** 1 Department of Cardiology, Functional and Ultrasound Diagnostics, Sechenov First Moscow State Medical University (Sechenov University), Moscow 119435, Russia; 2 Institute of Personalized Cardiology, Sechenov First Moscow State Medical University (Sechenov University), Moscow 119435, Russia

Coronary artery disease (CAD) is the leading cause of morbidity and mortality in developed and in most developing countries. Up-to-date guidelines are essential for most patients with all forms of CAD, including acute coronary syndromes (ACS) and chronic coronary syndromes (CCS). For most patients, we have powerful knowledge about diagnostic pathways, treatment logistics, indications for endovascular interventions or bypass surgery, inpatient treatment, and prevention. However, the prognosis for many patients is still poor.

Personalized medicine, a medical practice that assesses an individual patient’s characteristics, mainly genetics, proteomics, or imaging analysis, for decision making, could be one of the keys for filling in these gaps. The concept of personalized medicine is an element of the “P4” model of medicine: Predictive, Personalized, Preemptive, and Participatory, and could change current medical practice in the 21st century. This strategy accounts for individual differences in genetics, lifestyle, and other health factors in order to achieve in-depth disease phenotyping and to identify better clinical solutions for individual patients instead of using standard approaches. Personalized medicine requires big data analysis to generalize standard clinical and demographic variables to metabolomic and proteomic studies, novel biomarkers, and imaging technologies. Moreover, this type of analysis may implement artificial intelligence (AI) algorithms.

Instruments for personalized medicine have made a breakthrough, improving prognosis in many cancer patients. However, besides oncology, success has been modest. For example, in CAD, an analysis of the application of a pharmacogenomic approach prescribing antiplatelet and lipid-lowering drugs in clinical trials showed that the approach could not improve current treatment results effectively. However, the approach could decrease the rate of adverse events such as statin-related muscle disease. 

This Special Issue of the *Journal of Personalized Medicine* covers several questions about personalized medicine in CAD, with a special focus on the use of non-coning RNA as biomarkers or treatment targets and novel cardiac imaging modalities that can influence daily practice. 

A class of non-coding RNA, microRNAs (miRNAs), has been shown to be involved in a wide spectrum of biological or pathological mechanisms via the expression or translation of messenger RNA. MiRNAs have been found to be expressed in different tissues and can be measured in several body fluids, including serum.

These circulating miRNAs have increased the interest of researchers due to their stability in serum and availability for analysis. Several miRNAs have been proposed as circulating biomarkers for all stages of CAD, from primary prevention stratification and atherosclerosis identification to myocardial infarction diagnostics. 

Several circulating miRNAs have been proven to be biomarkers of myocardial infarction. Moreover, some circulating miRNAs can serve as destabilization markers of the atherosclerotic process and, accordingly, as early precursors of cardiovascular catastrophes.

The evaluation of microRNA levels makes it possible to indicate not only the presence of an atherosclerotic process, but also the severity of its course. Some circulating microRNAs are associated with the volume and extent of atherosclerotic lesions, while other microRNAs are correlated with the degree of arterial calcification.

Tissue and circulating miRNAs can maintain the vulnerability or stability of atherosclerotic plaques, contributing to a personalized treatment approach.

The regulatory function of microRNAs is so finely tuned that the levels of some of them can suggest a risk of disease symptoms developing or a disease’s asymptomatic course. Additionally, via the expression of some miRNAs, it is possible to assess which arterial regions are most susceptible to atherosclerotic processes.

It should be noted that several miRNAs are present in various components of the lipid spectrum and that their assessment can be used as an additional diagnostic and therapeutic tool in the field of complex dyslipidemias.

Tailoring prevention or intervention methods to the current disease, condition, and circumstances of individual patients is the basis of personalized medicine. Currently, we see the development of the following trends: the deepening of diagnostic and treatment methods at the molecular level and the acquisition of new information with very complex processing methods for available archived data. The possibility of using complex statistical models, mathematical modeling, and neural networks use make it possible to provide next-level decision support systems for use in clinical practice in the field of therapy selection or risk stratification for patients with coronary heart disease. Around the world, there are several ready-made or up-and-coming projects based on predictive models that are aimed specifically at patients with coronary artery disease and that allow for the personalized selection of therapy to determine a prognosis (ML4CAD, CoroPrevention, etc.).

New image processing methods for use in patients with coronary artery disease, such as personalized mathematical models of coronary blood flow, deserve special attention. This research direction has undergone significant development in recent years, with developments such as CT-FFR QFR (quantitative flow ratio). QFR uses 3D coronary and computational fluid dynamics to estimate fractional flow reserve based on angiography reconstruction alone. Computer and mathematical capabilities have made it possible to apply this technology directly during intervention on the heart (FAVOR III China).

In the near future, such tools will be able to make the transition from the “stratified medicine” approach that is widely used in cardiology (identification of a group of people with certain characteristics) to a full justification of prevention and treatment based on individual patient characteristics possible.

